# Oxidation of Quercetin and Kaempferol Markedly Amplifies Their Antioxidant, Cytoprotective, and Anti-Inflammatory Properties

**DOI:** 10.3390/antiox12010155

**Published:** 2023-01-09

**Authors:** Hernán Speisky, María Fernanda Arias-Santé, Jocelyn Fuentes

**Affiliations:** Laboratory of Antioxidants, Nutrition and Food Technology Institute, University of Chile, El Líbano 5524, Macul, Santiago 7810000, Chile

**Keywords:** flavonoid oxidation, antioxidants, benzofuranones, quercetin, kaempferol

## Abstract

The contention that flavonoids’ oxidation would necessarily lead to a loss of their antioxidant properties was recently challenged by the demonstration that quercetin oxidation leads to the formation of 2-(3,4-dihydroxybenzoyl)-2,4,6-trihydroxy-3(2H)-benzofuranone (Que-BZF), a metabolite whose antioxidant potency was notably higher than that of its precursor. Here, we compared and expanded the former observation to that of the quercetin analogue kaempferol. Oxidation of kaempferol led to the formation of a mixture of metabolites that included the 2-(4-hydroxybenzoyl)-2,4,6-trihydroxy-3(2H)-benzofuranone (Kae-BZF). Following the chromatographic isolation of Kae-BZF from such a mixture, its antioxidant, mitochondria- and cell-protecting, and NF-kB-inhibiting effects were assessed, and compared with those of Que-BZF, in Caco-2 cells exposed to indomethacin as a source of ROS. The concentrations of Que-BZF (100 nm) and Kae-BZF (1 nm) needed to attain their maximal protection effects were 50- and 5000-fold lower than those of their respective precursors. The former differences in concentrations were also seen when the abilities of Que-BZF and Kae-BZF to inhibit the indomethacin-induced activation of NF-kB were compared. These data not only reveal that the oxidative conversion of quercetin and kaempferol into their respective 2-benzoyl-2-hydroxy-3(2H)-benzofuranones (BZF) results in a considerable amplification of their original antioxidant properties, but also that the in the case of kaempferol, such amplification is 100-fold greater than that of quercetin.

## 1. Introduction

Quercetin and kaempferol are among the most ubiquitous and abundant flavonoids in edible plants [1,2]. Although these compounds display a wide range of bioactivities [3,4,5], their ability to act as antioxidants, namely, as molecules capable of lowering the intracellular formation of ROS and/or favoring their removal, constitutes the bioactivity most commonly associated with their health effects [6,7,8]. Although certain ROS play an essential role as physiological signaling molecules [9], avoiding their indiscriminate overproduction and/or intracellular accumulation is needed to prevent the development and/or progression of a large number of cardiovascular, neurodegenerative, and cancer diseases [10,11]. Like other flavonoids, quercetin and kaempferol can exert their ROS-removing effects directly, scavenging these species [12,13], or indirectly, upregulating the expression of genes that encode the synthesis of ROS-removing and antioxidant-synthesizing enzymes [14,15,16].

**Figure 1:**
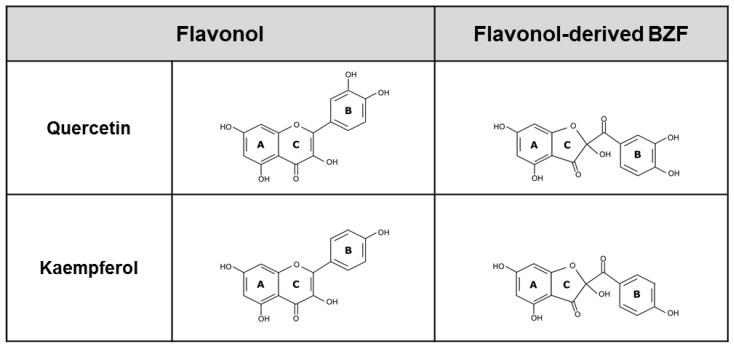
Chemical structures of the flavonols quercetin (3,3′,4′,5,7-pentahydroxyflavone) and kaempferol (3,4′,5,7-tetrahydroxyflavone), and their respective benzofuranones: quercetin-derived BZF (2-(3,4-dihydroxybenzoyl)-2,4,6-trihydroxy-3(2H)-benzofuranone), and kaempferol-derived BZF (2-(4-hydroxybenzoyl)-2,4,6-trihydroxy-3(2H)-benzofuranone).

As flavonols (FL), quercetin (Que) and kaempferol (Kae) share as a structural backbone a flavan nucleus that typically contains a double bond in C2−C3, a hydroxyl moiety in C3, and a keto group in C4 of ring C (Figure 1). They also share, in part, the pattern of benzene ring hydroxylation, as their structures include a hydroxyl group in C5 and C7 of ring A, and in the C4′ position of ring B. The only difference between Que and Kae is that the former exhibits an additional hydroxyl group in ring B, at position C3′. The pattern of benzene hydroxylation (i.e., number and positions) is a major determinant of the ability of flavonoids to exert their antioxidant action [17,18]. Depending on the mechanism involved in such actions, flavonoids can undergo no changes in their phenolic structure or be chemically modified in a manner that could severely affect their original antioxidant properties [19,20,21]. In general, when directly exposed to ROS, flavonoids undergo a partial or total loss of their antioxidant properties, especially of their ROS-scavenging/reducing property since the latter fully depends on the integrity of their redox-active phenolic moieties [22,23,24]. Recently, however, the contention that flavonoid oxidation would necessarily lead to a loss of their antioxidant properties was challenged by a study by Atala et al. [25] where, after inducing the total oxidation of various flavonoids in alkali, the mixtures of metabolites formed were found to exhibit antioxidant potencies that, in some cases, were equal or even superior to those of their precursors. Of particular interest was the case of quercetin, whose alkali-induced oxidation generates a mixture of metabolites, of which the major one was 2-(3,4-dihydroxybenzoyl)-2,4,6-trihydroxy-3(2H)-benzofuranone (Que-BZF, Figure 1). Isolation and extensive testing of Que-BZF in human intestinal epithelial cells (Caco-2) exposed to different ROS led to establishing that this metabolite affords full antioxidant protection at surprisingly low nanomolar concentrations [26]. Compared to quercetin, Que-BZF was found to exhibit three orders of magnitude higher antioxidant potency [18]. Interestingly, the only structural change associated with the oxidative conversion of quercetin into Que-BZF is that in the 2-benzoyl-2-hydroxy-3(2H)-benzofuranone (BZF) structure, the original six-atom pyran ring of the flavonol is transformed into a five-atom furanone ring to which a hydroxyl and a carbonyl group are bound (Figure 1). It should be noted that the studies conducted by Fuentes et al. [26,27] have been pioneers in demonstrating that the oxidative conversion of Que into its BZF metabolite could translate into an enormous “amplification of its original antioxidant potency”. Prompted by such studies, and by the extreme structural closeness of quercetin and kaempferol, the present study addressed the occurrence of the BZF derived from Kae, 2-(4-hydroxybenzoyl)-2,4,6-trihydroxy-3(2H)-benzofuranone (Kae-BZF, Figure 1), in a mixture of metabolites that result from its alkali-induced oxidation, and comparatively investigated the consequences that the formation of such metabolite could have in terms of its capacity to protect Caco-2 cells against the oxidative, mitochondrial, and cytolytic damage induced by indomethacin as an ROS-generator [28]. In addition, the potential of Kae-BZF to protect against the pro-inflammatory effects associated with the nuclear factor kappa B (NF-kB)-activating effects of indomethacin [29] was investigated.

## 2. Materials and Methods

### 2.1. Chemicals

Quercetin and kaempferol were purchased from Sigma-Aldrich (St. Louis, MO, USA), with a purity ≥ 95%. Acetonitrile, ethanol, and acetic acid were HPLC grade. Formic acid, sodium hydroxide, indomethacin, sodium acetate, sodium dihydrogen phosphate, disodium hydrogen phosphate, 2′,7′-dichlorodihydrofluorescein diacetate (DCFH2-DA), 3-(4,5-dimethyl-thiazol-2-yl)-2,5-diphenyltetrazolium bromide (MTT), and dimethyl sulfoxide were from Sigma-Aldrich. All other reagents were of analytical grade.

### 2.2. Preparation and Analysis of the Flavonol Oxidation Mixtures

Quercetin and kaempferol were initially dissolved in ethanol and, upon total dissolution, brought to a 10 mM concentration by the addition of sodium phosphate buffer (25 mM, pH 7.4). Prior to the addition of these flavonoids to the cells, solutions of these were diluted further with the same buffer to a level such that the cells were never exposed to a concentration of ethanol greater than 0.05% *v/v*. To obtain the oxidized mixtures of Que and Kae, the above-referred 10 mM FL solutions were added to NaOH (to reach pH 12) and, depending on the FL, incubated at 22 or 40 °C for different lengths of time. In each case, the incubation time was extended until obtaining a remnant concentration of 2% or less of that initially added. The concentration of FL in each oxidation mixture was monitored along the incubations and determined by HPLC using an Agilent 1200 series bomb, equipped with an autosampler and a photodiode array detector (Santa Clara, CA, USA). The HPLC system was controlled by Agilent ChemStation (Agilent Technologies 2010). The mobile phase consisted of a mixture of (A) acetonitrile and (B) 0.1% aqueous formic acid, whose composition was varied by employing the following HPLC gradient program: 0–15.0 min, 10% A, and 15.0–50.0 min, 10–60% A, and returned to starting conditions in the following 10 min. The column used was a 250 × 4.6 mm column, i.e., 5 μm, Kromasil 100-5-C18 (AkzoNobel, Bohus, Sweden). Other chromatographic conditions were as follows: flow rate of 0.8 mL/min and oven column set at 25 °C. The absorbance of the eluates was monitored at 370 nm for quercetin and 368 nm for kaempferol. The concentration of each FL was estimated from the area under the curve of its chromatographic peak using standard curves prepared just before the experiment. The standard deviation of each determination was always less than 5%.

### 2.3. Chromatographic Assessment, Identification, and Isolation of 2-Benzoyl-2-Hydroxy-3(2H)-Benzofuranones in the Flavonol-Oxidized Mixtures

Oxidation mixtures of Que (QueOx) or Kae (KaeOx), containing nearly 2% of the initial concentration of the FL, were subjected to HPLC analysis using chromatographic conditions identical to those described in Section 2.1. To assess the presence of BZF in such mixtures, the eluates were monitored at 290 nm. Since BZF are more polar than their respective flavonols, particular attention was placed on those peaks whose eluting times (tR) were shorter than those of their precursors. The second and more fundamental criterion to select a putative BZF peak was that its UV absorption spectrum corresponded with that described for it by Jungbluth and Ternes [30]. Subsequently, the eluting fraction corresponding to the selected BZF peak was collected using an automated fraction collector (Agilent 1260 Infinity coupled to the above-referred HPLC), and brought to dryness immediately after, using a rotatory evaporator at 40 °C. To ascertain the chemical innocuousness of the drying process, a sample of the dried fraction was re-chromatographed, assessing its stability in terms of conservation of the tR, the area under the curve of the putative BZF, and the absence of new peaks. To confirm the chemical identity of the molecule present in the latter, a sample of this was subjected to UHPLC-ESI-MS/MS analysis using a Shimadzu (Kyoto, Japan) Nexera X2 UHPLC system consisting of an LC-30AD pump, a DGU-20A5R degassing unit, a SIL-30AC autosampler, a CTO-20AC column oven, a CBM-20A communication module, an SPD-M20A diode array detector (DAD), and an LCMS-8030 triple quadrupole mass spectrometer. Data were acquired and recorded by means of Shimadzu LabSolutions version 5.51 software. Flow rates, HPLC column/oven column, and mobile phase composition/gradient were identical to those described in Section 2.2. The electrospray ionization (ESI) interface was operated in negative mode with a capillary voltage of 4.5 kV, desolvation line temperature of 250 °C, heat block temperature of 400 °C, nebulizing gas (N_2_) of 3.0 L min^−1^, drying gas (N_2_) of 15.0 L min^−1^, and collision gas (Ar) of 230 kPa. The collision energy was optimized for all compounds at 20 V and data acquisition was obtained in single-ion monitoring mode and multiple reaction mode.

### 2.4. Chemical Subtraction of 2-Benzoyl-2-Hydroxy-3(2H)-Benzofuranones from Flavonol-Oxidized Mixtures

The 2-benzoyl-2-hydroxy-3(2H)-benzofuranones present in the mixture of oxidation of Que or Kae were subjected to a chromatographic separation (using conditions identical to those described in Section 2.2) by means of an HPLC-DAD coupled to the fraction collector. To obtain preparations of flavonol-oxidized mixtures devoid of BZF, fractions of the whole chromatographic eluate, except for that corresponding to the BZF-containing peak, were collected, pooled, and brought to dryness using a rotary evaporator at 40 °C. A similar chromatographic procedure that did not exclude the collection of the BZF fraction was followed to obtain a dried preparation of each whole oxidation mixture. These dried preparations were stored at −80 °C for a maximum of one week. To ascertain that each of these dried preparations preserved its original chromatographic features (in terms of the number of peaks and their tR), they were subjected to HPLC-DAD analysis.

### 2.5. Cell Culture and Assessment of Oxidative Status and Antioxidant Effects

The antioxidant effects of the FL, their oxidation mixtures, and their corresponding BZF were assessed in Caco-2 cells exposed to indomethacin. Caco-2 cells, a human colonic adenocarcinoma cell line, have been broadly used as a model to study the oxidative and cytotoxic effects caused by indomethacin and other non-steroidal anti-inflammatory drugs on intestinal epithelial cells [28,29,31,32]. Caco-2 cells (ATCC^®^ HTB-37™), between the 10th and 15th passages, were cultured at 37 °C (5% CO_2_/95% air) in DMEM (Dulbecco’s Modified Eagle’s medium) supplemented with 10% fetal bovine serum (Biological Industries USA, Inc., Cromwell, CT, USA). Cells were trypsinized when they reached near 90% confluence and were used for experiments in which their intracellular oxidative status was assessed. As previously described by Fuentes et al. [26], DCFH2-DA was used as a ROS-reactive probe. The cells were loaded with DCFH2-DA (50 μM) for 30 min, and after being washed with PBS, the cells were incubated for 40 min in indomethacin (325 μM) in the absence or presence of either the FL, their oxidation mixtures, or their corresponding BZF. At the end of the incubations, cells were subsequently washed with PBS and lysed with the addition of Triton X-100 (0.03%). After 10 min, the cellular fluorescence was measured (excitation 495 nm/emission 529 nm) using a Synergy 2 multimode reader (Biotek, Winooski, VT, USA). The fluorescence value obtained after incubating Caco-2 cells with only PBS was used as the basal oxidative tone value. The over-the-basal increase in fluorescence observed in indomethacin-added cells was ascribed to a 100% value. The antioxidant effects of adding the FL, their oxidative mixtures, or their BZF to the cells were expressed as a percentage of the inhibition of the increase in fluorescence induced by indomethacin.

### 2.6. Assessment of Mitochondrial and Cytolytic Damage

Lactate dehydrogenase (LDH) leakage was used as a marker of cell lysis, and MTT reduction was used as a marker of mitochondrial viability. LDH was evaluated in the supernatants of cells exposed for 40 min to either PBS or indomethacin, in the absence or presence of FL, their oxidation mixtures, or their corresponding BZF, using the CytoTox-OneTM homogeneous membrane integrity assay (excitation 560 nm/emission 590 nm), carried out as previously reported [26]. The results are expressed as a percentage of LDH leaked into the extracellular media. MTT reduction into formazan, which evaluates the mitochondrial dehydrogenase activity, was evaluated in the adherent viable cells. After washing with PBS, 20 μL of 2.5 mg/mL MTT and 80 μL of PBS were added to each well, and cells were further incubated for 150 min at 37 °C (5% CO_2_/95% air). The supernatant was discarded, and 100 μL of dimethyl sulfoxide was added to dissolve the formazan. After 10 min of incubation at 37 °C, the absorbance at 540 nm was measured [27]. Results are expressed as the percentage of MTT reduction. A 100% MTT reduction was estimated in the PBS-treated cells.

### 2.7. Assessment of NF-kB Activity

Caco-2 cells (2 × 10^7^ cells/75 cm^2^ cell culture flask), incubated for 40 min with indomethacin in the absence or presence of FL, their oxidation mixtures, or corresponding BZF, were subjected to nuclear extraction using a Nuclear Extraction Kit (Cayman Chemical, Ann Arbor, MI, USA). Subsequently, NF-kB activation was evaluated using the NF-kB p65 (Total) ELISA Kit (Invitrogen^TM^, Carlsbad, CA, USA). Using pre-treated 96-well plates, samples of the nuclei extracts were incubated for 120 min. After discarding the supernatants, the plates were washed with a wash solution provided by the Kit, loaded with the NF-kB p65 (Total) detection antibody solution, and incubated for 60 min. Following removal of the supernatants and washing of the plates, these were loaded with anti-rabbit IgG HRP working solution and incubated for 30 min. After discarding the latter supernatant, the plates were washed, a stabilized chromogen solution was added, incubated under dark conditions for a 30 min period, and finally the Kit’s stop solution was added. The absorbance of the resulting solution was measured at 450 nm, using a standard curve provided by the Kit. Results are expressed as NF-kB p65 (ng/mg protein).

### 2.8. Statistical Analysis

Data were analyzed using the GraphPad Prism 5 statistical software (La Jolla, CA, USA). Values represent the means of at least three independent experiments, each conducted in octuplicate. Statistical significance of the differences between the experimental conditions was assessed with the analysis of variance (ANOVA) and post hoc Bonferroni test.

## 3. Results and Discussion

### 3.1. Time-Course Studies on the Chemical Oxidation of Flavonols

**Figure 2:**
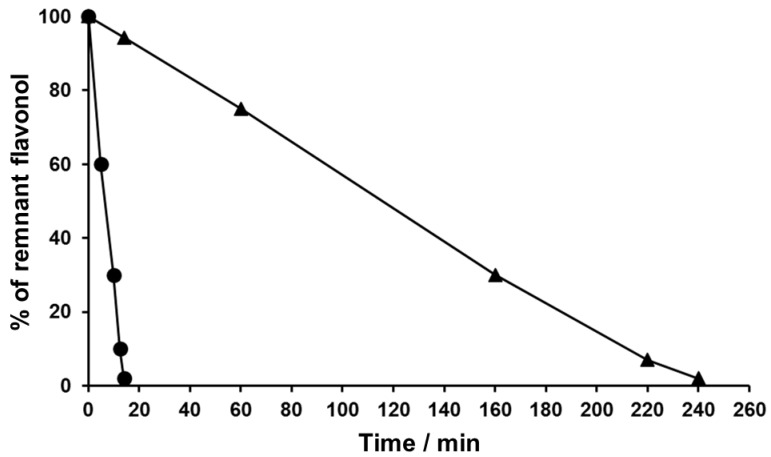
Time-dependent disappearance of quercetin and kaempferol following their chemical oxidation. After the initial dissolution of each flavonol in alkali medium, samples incubated for various durations (0–240 min) were neutralized and immediately assayed by HPLC-DAD, as described in the Materials and Methods Section. For each flavonol, data are presented as the remnant percentages of the initial concentration. The figure shows the decay profiles of quercetin (●) incubated at 22 °C, and of kaempferol (▲) incubated at 40 °C.

Figure 2 depicts the time-course of the oxidative disappearance of quercetin and kaempferol following their incubation in alkali medium at 22 °C. In the case of quercetin, its remnant concentration was less than 2% after 14 min. The latter value is almost identical to that previously reported by Atala et al. [25]. Since at 22 °C the concentration of kaempferol was only lowered by 50% after 100 min, its oxidative disappearance was accelerated by exposing it to 40 °C, a temperature at which its near-total disappearance was attained after 240 min. The mixtures of metabolites formed under conditions leading to a near-total disappearance of these flavonols will be referred to hereafter as FLox, in the case of quercetin as QueOx, and as KaeOx for kaempferol. Considering that both flavonols share the same number and position of hydroxyl groups in their A ring, the considerably slower oxidation of kaempferol, whose B ring features only a single hydroxyl (4′), suggests that the presence of a catechol group (3′,4′) in such ring underlies the notably higher oxidation of quercetin. In fact, the first step in the oxidation of a catechol flavonoid involves the initial removal of an electron or a hydrogen atom from one of its hydroxyl groups to generate an o-semiquinone intermediate whose stability depends on the probability of hydrogen bonding with the oxygen of a vicinal hydroxyl group [33].

### 3.2. Antioxidant Effects of Quercetin and Kaempferol, and Their Respective Oxidation Mixtures

**Figure 3:**
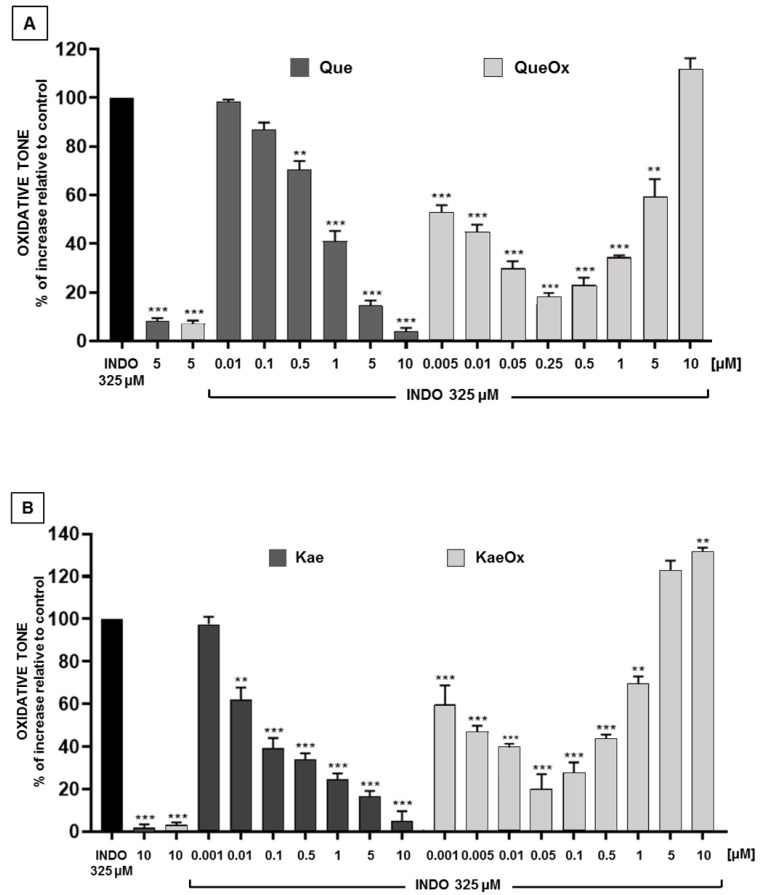
Comparison of the antioxidant effects of quercetin and kaempferol and their respective oxidation mixtures against the increase in intracellular oxidative tone induced by indomethacin. (**A**) Que versus QueOx, and (**B**) Kae versus KaeOx. Significant differences: ** *p* < 0.01, and *** *p* < 0.001 relative to indomethacin-treated cells.

The antioxidant effects of quercetin and kaempferol, and that of their respective FLox against the increase in oxidative tone induced by indomethacin in Caco-2 cells, are described in Figure 3. In the case of the non-oxidized flavonols, the antioxidant effects followed a concentration-dependent pattern. Quercetin and kaempferol afforded a near 80% protection at 5 μM. In the case of their FLox, a concentration dependence was also seen. It should be noted, however, that the concentrations of FLox described in Figure 3 (and in subsequent figures) correspond to “equivalent concentrations”, namely, concentrations whose values were estimated based on the dilution of the concentration of the FL that was initially subjected to oxidation. Accordingly, a near 80% protection against the increase in oxidative tone was observed at equivalent concentrations of 0.25 μM for QueOx and 0.05 μM for KaeOx. The latter concentrations evidence that inducing the total oxidation of Que or Kae results in mixtures of metabolites that differ in terms of their composition and whose antioxidant properties are greater than those of their precursors. In each case, the increase in such properties is manifested by the substantial dilution that the FLox had to undergo to generate a degree of protection similar to that afforded by the FL. While in the case of Que the oxidation led to a 20-fold higher antioxidant activity (Que 5 μM versus QueOx 0.25 μM), that associated with the total oxidation of Kae was 100-fold (Kae 5 μM versus KaeOx 0.05 μM). These increases should be interpreted only as an indication of the existence of differences in the antioxidant potency of each FL and its FLox. In addition, it should be noted that for a given FLox, the increase in antioxidant properties will strictly depend on the initial concentration of the FL and the experimental conditions that have led to its oxidation, such as the temperature and time of exposure, and the nature of the oxidizing environment. The antioxidant protection afforded by the FLox was concentration-dependent, being manifest within the concentration ranges of 0.005–0.25 μM for QueOx and 0.001–0.05 μM for KaeOx. Interestingly, Figure 3 also shows that the efficiency of such protections starts to decay when the oxidation mixtures are tested at equivalent concentrations higher than those needed to attain their maximum effects. The latter effect is evident for QueOx, when tested within the 0.5–5 μM range, and for KaeOx within the 0.1–1 μM range. Moreover, when QueOx and KaeOx were tested at concentrations superior to such ranges, these not only failed to exert any protection but raised the oxidative tone of the cells to a level higher than that induced by indomethacin. The U-shaped-type behavior of these FLox could be interpreted as an indication that within their composition, one or more molecules would be included that, within a low range of concentrations, are able to trigger antioxidant effects, but that beyond the concentrations at which the FLox afford their maximal antioxidant effects, such molecules would start to behave as pro-oxidants. This biphasic mode of action, where opposite effects take place at a different range of concentrations, is referred to as a para-hormetic behavior [34,35] and has been described for a large number of phytochemicals [36]. Regarding the nature of the molecules that might underlie the 20- and 100-fold increases in antioxidant potency seen with QueOx and KaeOx, respectively, we investigated the possible presence of 2-benzoyl-2-hydroxy-3(2H)-benzofuranones in these mixtures.

### 3.3. Chromatographic Assessment of the Presence of 2-Benzoyl-2-Hydroxy-3(2H)-Benzofuranones in the Quercetin and Kaempferol Oxidation Mixtures

**Figure 4:**
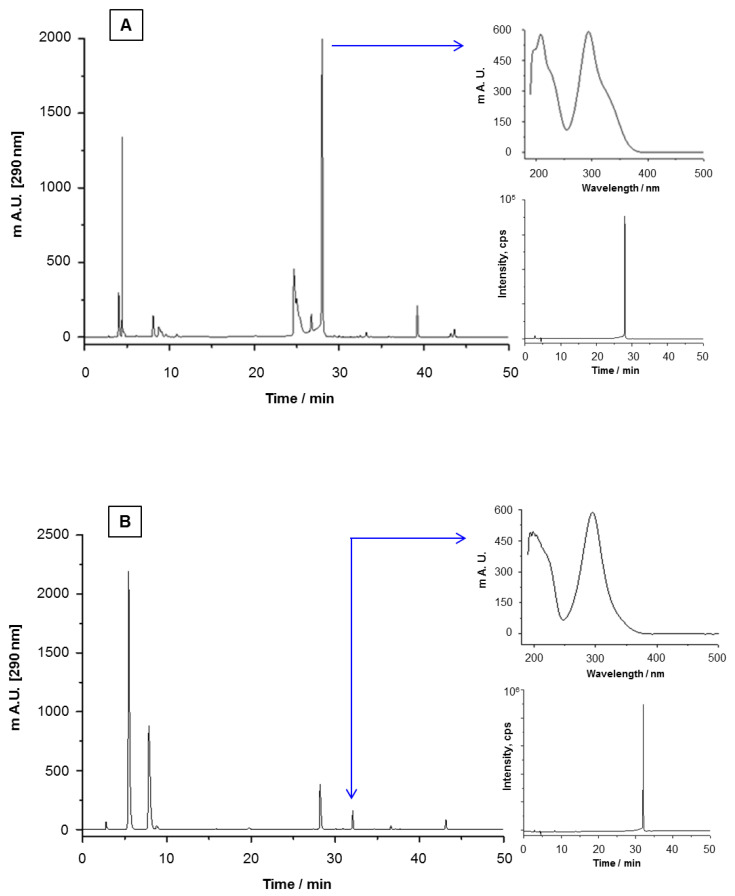
Chromatograms resulting from HPLC-DAD analyses of the oxidation mixtures of the flavonols: (**A**) quercetin and (**B**) kaempferol. In each chromatogram, the peak corresponding to the BZF is indicated with an arrow that connects it with its corresponding UV absorption spectrum. In addition, the UHPLC-ESI-MS/MS chromatograms of the BZF isolated from each oxidation mixture are presented.

To search for the presence of BZF in the mixtures of alkali-induced oxidation of the flavonols under study, samples of their FLox were subjected to HPLC-DAD analysis using the wavelength reported by Jørgensen et al. [37]: 290 nm for quercetin and kaempferol. Although the chromatograms of FLox depicted in Figure 4 evidence the presence of various peaks, we focused and limited our search only to those whose UV absorption spectrum corresponded to the ones described by Jørgensen et al. [37] for these BZF. On the left side of Figure 4, parts A and B, the peaks of the putative BZF of quercetin and kaempferol are signaled by an arrow in each chromatogram, respectively. The right upper side of Figure 4 shows the UV absorption spectra of the signaled peaks. In each case, the spectrum obtained is almost identical to those reported after inducing their electrochemical [37] or metal-catalyzed [30] formation. Following this chromatographic peak selection criteria, the peak corresponding to each putative BZF was isolated from its corresponding FLox and subjected to UHPLC-ESI-MS/MS analysis. As shown in the right lower side of parts A and B of Figure 4, the tR of these analyses were identical to those of the putative BZF peaks seen after subjecting the FLox to HPLC-DAD analysis. Retention times of 27.8 and 31.9 min were obtained for the BZF of quercetin and kaempferol, respectively. For each of these BZF, whose chemical structures are depicted in Figure 1, the molecular ion [M-H]- and corresponding qualification transitions were *m*/*z* at 317.0 and 163.1/191.0 for Que-BZF and *m*/*z* at 301.1 and 245.1/273.1/151.1/206.9 for Kae-BZF. These data are almost identical to those reported by Zhou and Sadik [38] and Fuentes et al. [26] for the Que-BZF, and by Jungbluth and Ternes [30] for the Kae-BZF. The former results not only confirm the presence of the BZF of quercetin and kaempferol in their corresponding FLox but also reveal that for kaempferol, the formation of its BZF metabolite can take place under alkaline conditions. Faced with the question of whether the higher antioxidant properties of the FLox described in Figure 3 are attributable to the presence of BZF in their composition, we pursued chemical subtraction studies in which the latter metabolites were specifically removed from their oxidation mixtures.

### 3.4. Antioxidant Effects of BZF-Containing and BZF-Free Oxidation Mixtures

**Figure 5:**
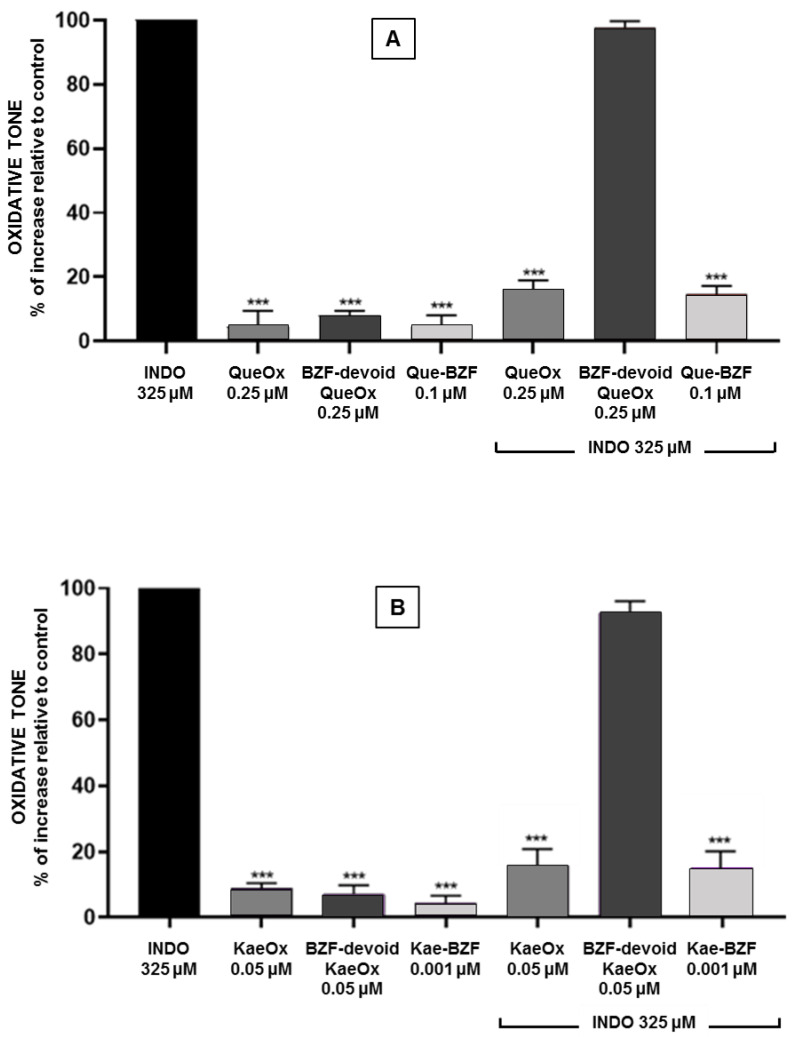
Comparison of the antioxidant effects of BZF-containing versus BZF-devoid flavonol oxidation mixtures. The antioxidant effects of QueOx and KaeOx are, in each case, compared with those elicited by the oxidation mixtures of (**A**) quercetin and (**B**) kaempferol, from which the BZF component was removed by chemical subtraction, and with those elicited by the BZF isolated from the former. In each case, the antioxidant effect was assessed against the increase in intracellular oxidative tone induced by indomethacin. Significant differences: *** *p* < 0.001 relative to indomethacin-treated cells.

To assess the contribution of Que-BZF and Kae-BZF to the antioxidant effects displayed by their corresponding FLox, samples of the latter were subjected to a chromatographic separation using an HPLC coupled with a fraction collector. These studies were conducted to obtain three different preparations. One consisting of a composition where the eluate corresponding to the total chromatographic run was collected and pooled (BZF-containing FLox), another where the same collection was carried out, except for the peak corresponding to the BZF (BZF-devoid FLox), and finally, one where only the peak corresponding to BZF was collected (isolated BZF). The eluate of each of these preparations was brought to dryness, reconstituted in PBS, and assessed for its ability to prevent the increase in oxidative tone induced by indomethacin in Caco-2 cells. As shown in Figure 5A, the protection afforded by the addition of BZF-containing QueOx at 0.25 μM (equivalent concentration) to the cells was totally lost when an identical BZF-devoid QueOx preparation was added. A similar situation was seen when a BZF-containing KaeOx preparation and its corresponding BZF-devoid preparation (Figure 5B), both added at 0.05 μM, were compared. These results strongly suggest that the higher antioxidant properties of these FLox are exclusively attributable to the presence of BZF in their composition. Further support for this contention was provided by the demonstration that the addition of isolated Que-BZF or Kae-BZF to the cells, at concentrations identical to those at which they are present in the BZF-containing FLox, led to a degree of protection similar to that afforded by the latter. Interestingly, in the absence of indomethacin, none of the three preparations induced a change in the basal oxidative tone of the cells.

### 3.5. Antioxidant Effects of Increasing Concentrations of Isolated 2-Benzoyl-2-Hydroxy-3(2H)-Benzofuranones

**Figure 6:**
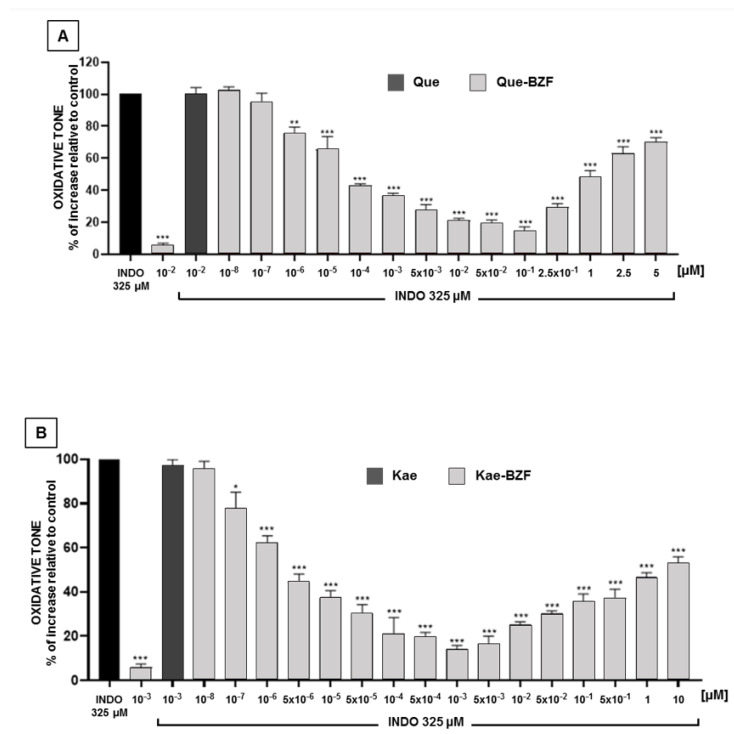
Antioxidant effects of increasing concentrations of BZF isolated from oxidation mixtures of (**A**) quercetin and (**B**) kaempferol, against the increase in intracellular oxidative tone induced by indomethacin. Significant differences: * *p* < 0.05, ** *p* < 0.01, and *** *p* < 0.001 relative to indomethacin-treated cells.

Having established that the higher antioxidant properties of each FLox arise from and fully depend on the presence of BZF in its composition, the possible concentration dependence of the antioxidant effects of BZF isolated from their respective FLox was investigated. Figure 6A,B describe that Que-BZF and Kae-BZF protect Caco-2 cells against the increase in oxidative tone induced by indomethacin in a concentration-dependent mode. Que-BZF afforded a near 80% protection (EC_80_) within the 10^−2^ μM and 5 × 10^−2^ μM range of concentrations. In the case of Kae-BZF, such a degree of protection was seen within the 10^−4^ and 5 × 10^−4^ μM range. These BZF also largely differ in terms of the concentrations at which they exert their maximal protection (near 90%). Such degree of protection was attained at 10^−1^ μM for Que-BZF and at 10^−3^ μM for Kae-BZF. Figure 6A,B also show that no protection was attained when quercetin or kaempferol was added to the cells at concentrations identical to those at which their respective BZF exert their maximal effects. Unlike the case of QueOx and KaeOx, whose relative antioxidant potencies cannot be compared because the concentrations at which they exert their maximal protections are only equivalent concentrations, in the case of Que-BZF and Kae-BZF, such comparison and the one relative to their respective FL was possible since the concentrations of the BZF were calculated on the basis of the molar extinction coefficients previously reported for each of these compounds [37]. When the concentration of Que-BZF needed to attain its maximal protection (10^−1^ μM, Figure 6A) was compared with the concentration of quercetin (5 μM) needed to afford such a degree of protection (data from Figure 3A), Que-BZF was found to be 50-fold more potent. In the case of Kae-BZF, the antioxidant potency was estimated to be 5000-fold higher than that of its precursor (10^−3^ μM, from Figure 6B versus 5 μM, from Figure 3B). These antioxidant potency data reveal not only that the oxidation of the two flavonols results in a considerable amplification of their original antioxidant properties, but that in the case of Kae, such amplification is 100-fold greater than that of Que. We propose to use the term “antioxidant amplification index” as a quantitative measure of the gain in antioxidant potency that quercetin, kaempferol, or eventually another BZF-forming FL acquire when converted into their respective BZF. Within the frame of such conceptualization, a comparison between different FL could allow the establishment of rankings. Compared to quercetin, the substantially greater antioxidant amplification index of kaempferol is notable considering that the only structural difference between these two molecules (Figure 1) is that kaempferol lacks the hydroxyl group that quercetin exhibits in the C3 position of ring B. One may speculate that such moiety disfavors the antioxidant properties of the BZF, however, prior to such contention, one would need considerably more information about the antioxidant potencies of BZF derived from the oxidation of other flavonols. For instance, that derived from myricetin, which besides containing a hydroxyl group in C4 contains two other hydroxyls in positions C3 and C5 of ring B [30], and that derived from morin, which besides containing a hydroxyl group in C4 contains one in the C2 position of such ring [30]. On the other hand, it should be noted that since quercetin and kaempferol do not differ in their capacity to protect Caco-2 cells against the increase in oxidative tone induced by indomethacin, as shown in Figure 3, the eventual existence of differences in the antioxidant potencies that other FL might exhibit cannot be assumed as a predictor that such differences shall also be seen among their respective BZF.

Regarding the elevation of the oxidative tone induced by indomethacin in Caco-2 cells, such effect is believed to result primarily from the increase in ROS that results from the mitochondrial dysfunction induced by such agent, and that can ultimately lead to apoptosis [31,39,40]. Thus, in addition to assessing the antioxidant capacity of the FLox and BZF, the ability of these preparations to protect Caco-2 cells against mitochondrial-disturbing (i.e., MTT reduction-inhibition) and the cell lytic (i.e., LDH leakage) effects of indomethacin were evaluated. As shown in Figure S1A,B of the Supplementary Material, the basal leakage of LDH (corresponding to PBS-treated cells) was doubled by indomethacin. This effect was completely prevented when 5 μM of Que, 0.25 μM of QueOx (equivalent concentration), or 0.1 μM of Que-BZF were added to the cells. Identical protections were observed when 5 μM of Kae, 0.05 μM of KaeOx (equivalent concentration), or 0.001 μM of Kae-BZF were used. A similar protection pattern was seen when preparations of quercetin or kaempferol, added at the above-referred concentrations, were tested for their capacity to protect cells against the inhibition of MTT reduction induced by indomethacin (Figure S2A,B). It should be noted that the mitochondria and cell protective effects of Que-BZF and Kae-BZF were seen at the same concentrations at which these compounds exert their antioxidant effects, suggesting that the latter would underlie their mitochondria and cell protective effects [27,28]. In the case of Kae-BZF, such protections are seen at 0.001 μM, namely, at a remarkably low concentration, which suggests that the interaction between the Kae-BZF molecule and its biological target(s) takes place with a high degree of specificity. This would situate the Kae-BZF molecule as the, so far reported, more potent antioxidant molecule. The here-addressed ability of Kae-BZF and Que-BZF to protect against the oxidative and mitochondrial damage at such extremely low concentrations would warrant its future testing in other cells and disease models. Within such frame, worth nothing is the fact that ROS production and mitochondrial dysfunction have been closely implied in the development of neurodegenerative [41], cardiovascular [42], and cancer diseases [43].

**Figure 7:**
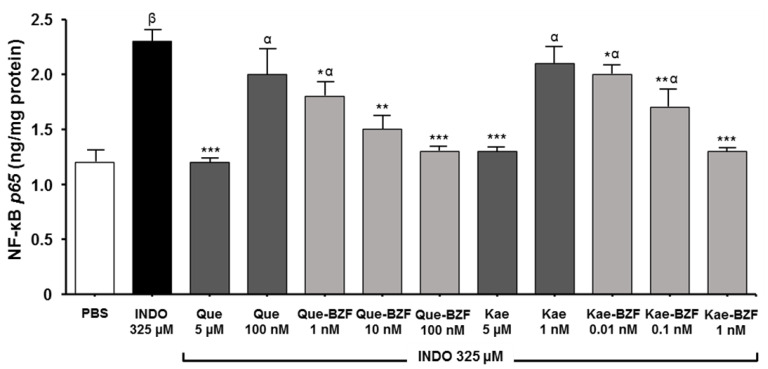
Effects of quercetin, kaempferol, and the respective BZF on Nuclear Factor kappa B activation by indomethacin. Caco-2 cells were incubated with INDO for 40 min in the presence of Que, Kae, Que-BZF, and Kae-BZF, and the NF-kB p65 subunit was assessed in the nucleus. Significant differences: * *p* < 0.05, ** *p* < 0.01, and *** *p* < 0.001 relative to INDO-treated cells, and α *p* < 0.01 and β *p* < 0.001 relative to PBS-treated cells.

Although the scope of the present study did not comprise addressing the mechanism(s) by which Kae-BZF exerts its protective effects, we focused our attention on the fact that indomethacin, in addition to inducing oxidative stress, induces the activation of the pro-oxidant and pro-inflammatory nuclear factor kappa B in Caco-2 cells [29], and intestinal mucosa of rats [44] exposed this agent. The activation of NF-kB can be both a cause and a consequence of the genesis of ROS [45]. Interestingly, Fuentes et al. [29] observed that, when the BZF derived from quercetin is added to Caco-2 cells at a 0.1 μM concentration, it completely prevents such activation. Considering the latter, the ability of Kae-BZF and its precursor to prevent the activation of NF-kB was evaluated and compared with that of Que-BZF. Data shown in Figure 7 confirm the ability of 0.1 μM of Que-BZF to completely prevent the activation of NF-kB. At this concentration, quercetin was unable to promote such an effect. However, a total protection was attained when quercetin was added to the cells at 5 μM. The figure also depicts that Kae-BZF is able to inhibit NF-kB activation by near 62% and 86% at 10^−4^ and 10^−3^ μM, respectively. At the latter concentration, kaempferol exerted no effect on NF-kB, but a 100% inhibition of its activation was seen at 5 μM. When the two BZF were compared, the maximal inhibitory effect of Kae-BZF on NF-kB was seen at a 100-fold lower concentration. Since the latter value does not differ from that emerging after comparing the concentrations of Kae-BZF and Que-BZF needed to attain their maximal antioxidant effects, it is tempting to speculate that the inhibition of the activation of NF-kB by these BZF could underlie their antioxidant effects. In view of the major role played by the activation of NF-kB in the development, maintenance, and progression of many inflammatory diseases [46,47], the here-reported extremely low concentrations of BZF needed to prevent its activation warrant further investigations on these molecules as potential antioxidant, cytoprotective, and anti-inflammatory agents.

## 4. Conclusions

The present study showed that the alkali-induced oxidation of quercetin or kaempferol led to the formation of mixtures of metabolites whose antioxidant and mitochondrial- and cell-protective properties were notably higher than those of their non-oxidized precursors. Chemical subtraction experiments led to the demonstration that, in each case, the increases in such properties were dramatic, and these would reside exclusively in the presence of the 2-benzoyl-2-hydroxy-3(2H)-benzofuranones of quercetin and kaempferol in such mixtures. Furthermore, in the case of Kae, the amplification of its antioxidant properties was found to be 100-fold greater than that of Que, revealing that after undergoing oxidative conversion into BZF, even a minor structural difference can cause unexpectedly large differences in the new antioxidant potencies of these two compounds. We propose that the here-presented data could open the possibility of defining the basic BZF skeleton as a top seed structure for the subsequent development and/or design of novel antioxidant molecules.

## Supplementary Materials

The following supporting information can be downloaded at: https://www.mdpi.com/article/10.3390/antiox12010155/s1, Evaluation of the capacity of quercetin, kaempferol and their respective FLox and BZF to protect cells against indomethacin-induced lytic and mitochondrial damage. Figure S1: Comparison of the capacity of (A) quercetin, its oxidation mixture, and its corresponding BZF, and that of (B) kaempferol, its oxidation mixture, and its corresponding BZF, to protect cells against the increase in lactate dehydrogenase leakage induced by indomethacin. Significant differences: * *p* < 0.05 and ** *p* < 0.01 relative to INDO-treated cells, and α *p* < 0.001 relative to the PBS-treated cells. Figure S2: Comparison of the capacity of (A) quercetin, its oxidation mixture, and its corresponding BZF, and that of (B) kaempferol, its oxidation mixture, and its corresponding BZF, to protect cells against the decrease in MTT reduction induced by indomethacin. Significant differences: *** *p* < 0.001 relative to INDO-treated cells, and α *p* < 0.001 relative to the PBS-treated cells.

## Abbreviations

BZF, 2-benzoyl-2-hydroxy-3(2H)-benzofuranone(s); DCFH2-DA, 2′,7′-dichlorodihydrofluorescein diacetate; FL, flavonol; FLox, flavonol oxidation mixture(s); INDO, indomethacin; Kae, kaempferol; Kae-BZF, 2-(4-hydroxybenzoyl)-2,4,6-trihydroxy-3(2H)-benzofuranone; KaeOx, kaempferol oxidation mixture; LDH, lactate dehydrogenase; MTT, 3-(4,5-dimethyl-thiazol-2-yl)-2,5-diphenyltetrazolium bromide; NF-kB, Nuclear Factor kappa B; Que, quercetin; Que-BZF, 2-(3,4-dihydroxybenzoyl)-2,4,6-trihydroxy-3(2H)-benzofuranone; QueOx, quercetin oxidation mixture; ROS, reactive oxygen species; tR, retention time.
