# Supplementary figures and images for: Oxidation of Quercetin and Kaempferol Markedly Amplifies Their Antioxidant, Cytoprotective, and Anti-Inflammatory Properties
Source: Antioxidants (Basel). 2023 Jan 9;12(1):155. doi: 10.3390/antiox12010155 (PMC9854986; doi:10.3390/antiox12010155)

## Slide 1
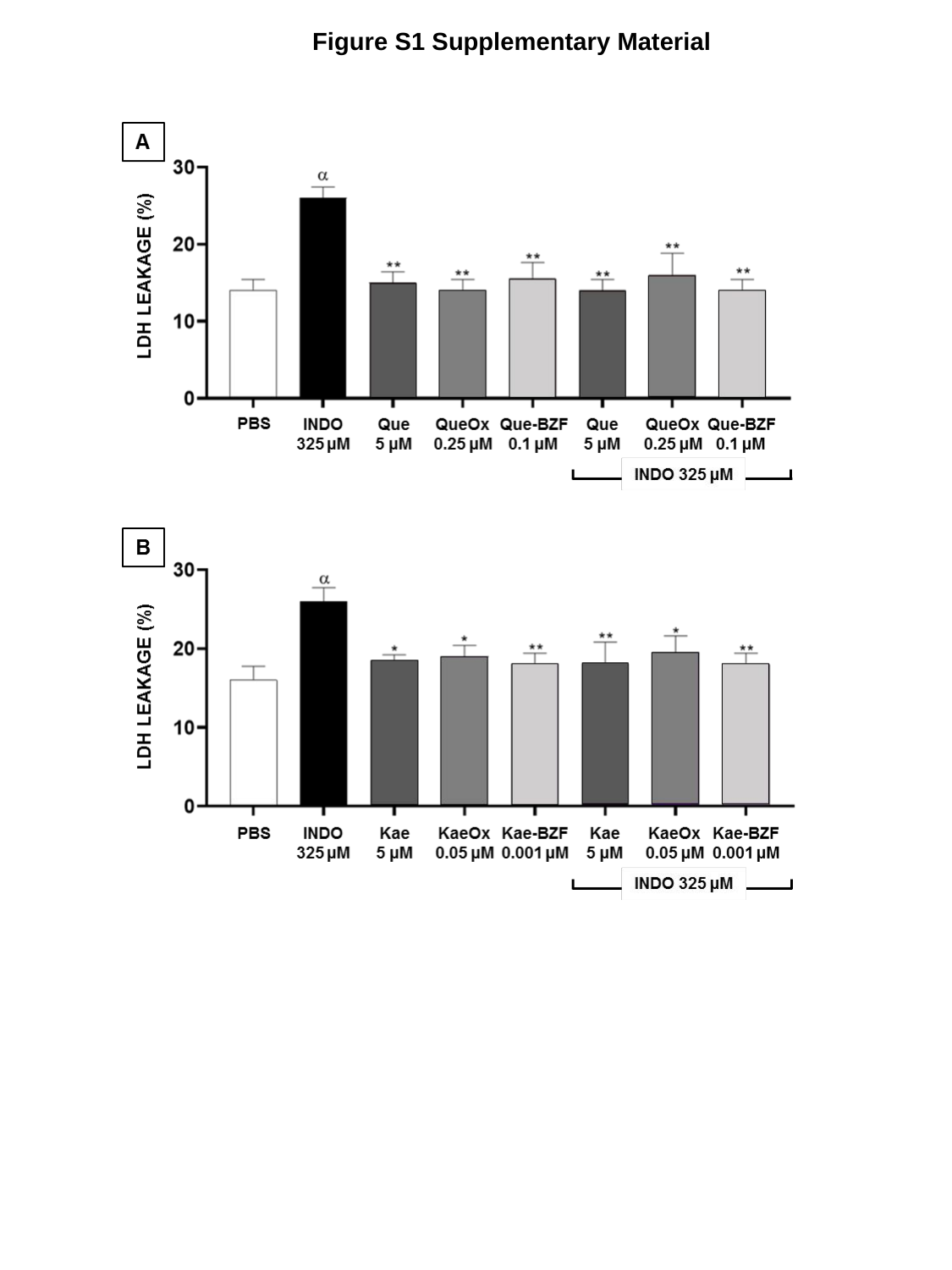

Figure S1 Supplementary Material

Supplement: Supplementary file 1 [file antioxidants-12-00155-s001.zip › SUPPLEMENTARY FIGURE S1-291122.pptx]

## Slide 1
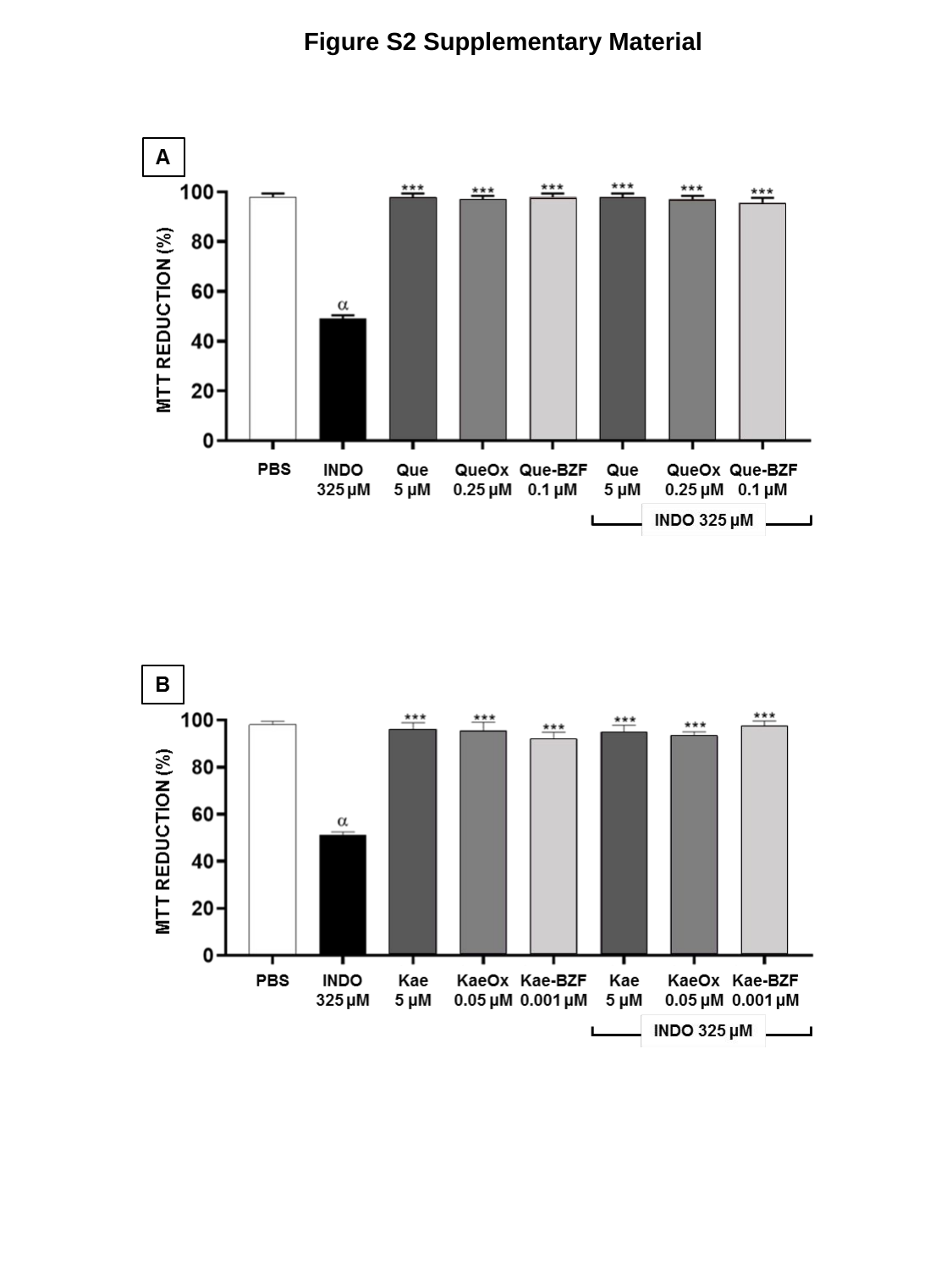

Figure S2 Supplementary Material

Supplement: Supplementary file 1 [file antioxidants-12-00155-s001.zip › SUPPLEMENTARY FIGURE S2-291122.pptx]
